# Fatal Disseminated Tuberculous Peritonitis following Spontaneous Abortion: A Case Report

**DOI:** 10.1155/2014/125609

**Published:** 2014-03-04

**Authors:** Munire Erman Akar, Tayfun Toptas, Havva Sutcu, Haney Durmus, Murat Ozekinci, Melike Cengiz, Gulgun Erdogan

**Affiliations:** ^1^Department of Obstetrics & Gynecology, Akdeniz University Hospital, 07070 Antalya, Turkey; ^2^Department of Anesthesiology and Reanimation, Akdeniz University Hospital, 07070 Antalya, Turkey; ^3^Department of Pathology, Akdeniz University Hospital, 07070 Antalya, Turkey

## Abstract

We describe a rare case of fatal disseminated tuberculous peritonitis in a young woman with rapid progressive clinical course following spontaneous abortion of 20-week gestation. Clinical and laboratory findings were initially unremarkable. She underwent diagnostic laparoscopy which revealed numerous tiny implants on the peritoneum and viscera. Histopathology showed chronic caseating granulomas, and the tissue culture grew Mycobacterium tuberculosis. At fifth day of the antituberculous treatment multiorgan failure occurred in terms of pulmonary, hepatic, and renal insufficiency. She developed refractory metabolic acidosis with coagulopathy and pancytopenia, and she died of acute respiratory distress syndrome and septic shock on her twelfth day of hospitalization.

## 1. Introduction

Tuberculosis (TB) is a major global health concern. The World Health Organization has declared TB a global emergency in 1994 [1]. Although it is a preventable and treatable disease, eight to ten million people develop TB every year; at least two million people die from this disease annually [2]. The incidence of the disease is 130 per 100000 people in the world and 22 per 100000 people in Turkey [3]. TB is a significant contributor to maternal mortality, with the disease being among the three leading causes of death among women aged 15–45 years [4]. Complications that have been reported in pregnancy include a higher rate of spontaneous abortion, small for date uterus, suboptimal weight gain in pregnancy, preterm labor, low birth weight, and increased neonatal mortality. Late diagnosis is an independent factor, which may increase obstetric morbidity about fourfold, while the risk of preterm labor may be increased ninefold [5].

Although the primary site for TB is lungs, one-third of patients might have extrapulmonary disease [6]. The peritoneum is one of the most common extrapulmonary sites of the disease. Disseminated or milier TB denotes all forms of progressive, widely disseminated hematogenous TB [7]. It can be primary fulminant including multiorgan system failure, septic shock, and acute respiratory distress syndrome (ARDS) with an acute onset and rapid clinical course or could be a reactivation of a latent focus, which is more likely to be subacute and chronic [7, 8]. Herein we report a rare case of fatal disseminated tuberculous peritonitis in a young woman with rapid progressive clinical course following spontaneous abortion of 20-week gestation.

## 2. Case Presentation

**Figure 1:**
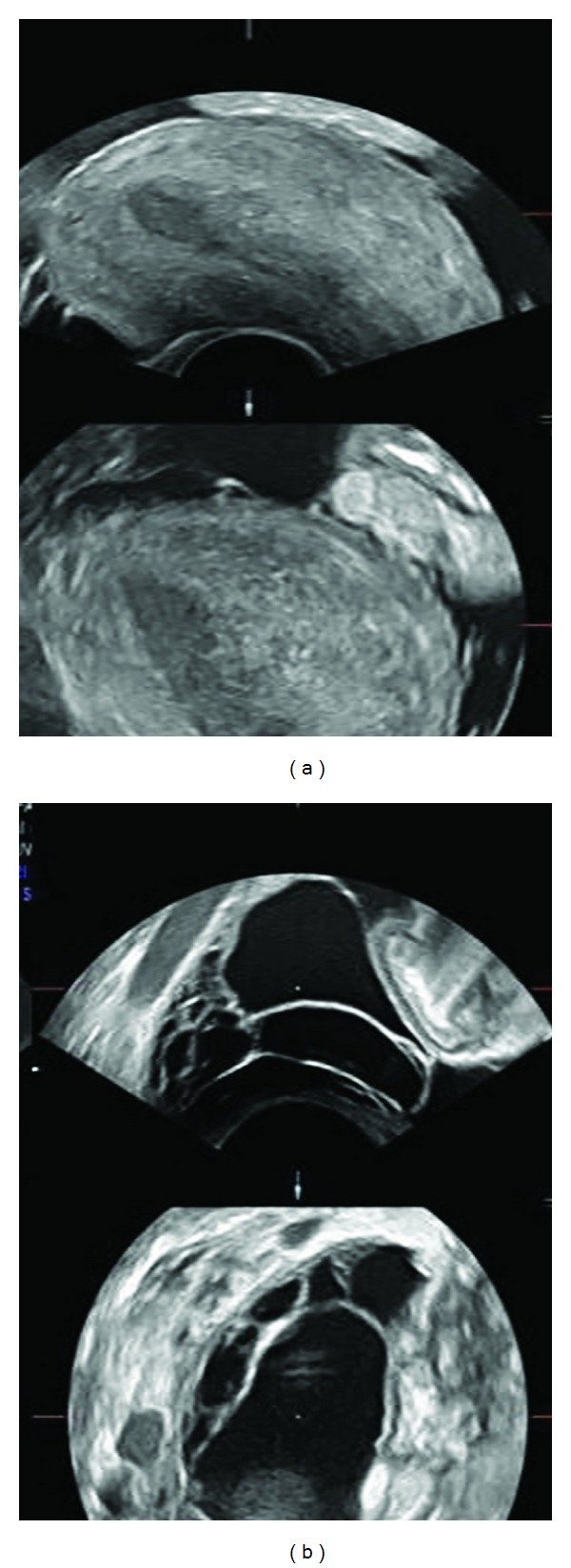
Transvaginal ultrasonography: (a) uterus and ascites; (b) hydrosalpinx.

A 25-year-old, gravidity 3, parity 1, spontaneous abortion 1, and medical abortion with suction curettage 1, woman referred to our clinic with diffuse abdominal pain of 2-week duration, massive ascites, and prolonged intermittent fever rising up to 40.0°C. She had suffered from a spontaneous abortion of 20-week gestation in another hospital nine days ago. She reported a lack of appetite, dizziness, sweating, and raised temperature persisting for at least 3 weeks. She denied cough or weight loss. On physical examination, she had an abdominal distension that suggested a fluid collection, diffuse tenderness especially in lower abdomen, and unremarkable uterine cervix with positive motion tenderness test. No signs of portal hypertension were present. Transvaginal ultrasound confirmed the presence of ascites and bilateral hydrosalpinx (Figure 1). Her hemoglobin, thrombocyte, C-reactive protein, and white blood count were 7.9 gr/dL, 112000 /mm^3^, 21.6 mg/L, and 8010 *μ*/L with low lymphocyte number, respectively. HIV test, serologies for hepatitis, and cultures of blood and urine were negative. Renal function tests and hepatic transaminase levels were in normal range. Serum carcinogenic antigen (Ca)-125 level was 669 Units/mL. Chest radiography revealed small pleural effusions bilaterally. The diagnostic paracentesis yielded inflammation with lymphocyte predominance and reactive mesothelial cells. Cytological analysis for atypical cells was negative. No acid-fast bacilli were seen by microscopy. Results of TB polymerase chain reaction (PCR) and culture of the ascitic fluid were negative. The patient was initially treated with intravenous broad-spectrum antibiotics, but medical treatment failed to improve her clinical condition, and she was further admitted to intensive care unit. Based on history and laboratory review, thoracic and abdominal computed tomography (CT) scans were obtained to further workup the etiology of the ascites. These studies demonstrated bilateral pleural effusion, atelectasis, pulmonary parenchymal frosted glass aspect, massive ascites, hepatomegaly, hepatosteatosis, and a pattern of peritoneal carcinomatosis with formation of an omental cake without definite adnexial tumoral mass. Upper gastrointestinal tract endoscopy revealed no pathologic findings. About 7 days after admission, the patient subsequently underwent diagnostic laparoscopy, which revealed massive ascites, extensive adhesions between viscera, and numerous tiny nodular implants on the peritoneal surfaces, liver, stomach, ovaries, intestine, omentum, and mesentery. Because of the presence of suspicion for malignancy, laparoscopy converted to exploratory laparotomy. Adhesiolysis was performed. The uterus was 6–8 weeks in size with nodular serosal surface. Bilateral fallopian tubes were grossly distended and were tortuous with unrecognizable fimbria. Multiple targeted biopsies were taken from the suspicious implants. Tissue samples from the fimbria, peritoneum, and omentum were sent for frozen section analysis and were found to be consistent with granulomatous inflammation. Some tissue samples and ascitic fluid were sent for the microbiologic examination.

**Figure 2:**
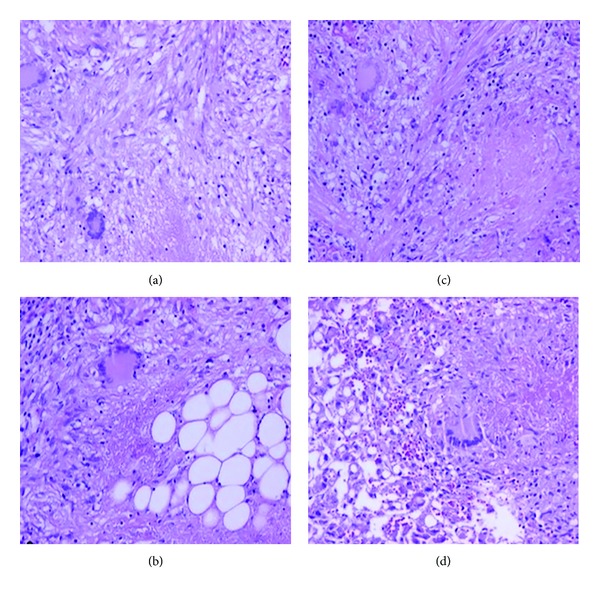
Chronic caseating granulomas with central necrosis: (a) fimbria; granuloma; and Langhans' giant cells (H&E, ×200), (b) peritoneum; granulomatous peritonitis; and Langhans' giant cell (H&E, ×200), (c) omentum; area of caseation between epithelioid histiocytes; lymphocytes; and Langhans' giant cell (H&E, ×200), (d) liver; steatosis; and Langhans' giant cell with area of caseation (H&E, ×200).

Postoperatively, antimycobacterial therapy with isoniazid, rifampin, pyrazinamide, and ethambutol was planned and started empirically. She was hypotensive and required vasopressors. Her blood gasses and control chest radiography which revealed diffuse pulmonary infiltrates were compatible with ARDS. The patient was sedated, and lung protective mechanical ventilation strategies were used. At fifth day of the antituberculous treatment multiorgan failure occurred in terms of pulmonary, hepatic, and renal insufficiency. She developed refractory metabolic acidosis with coagulopathy and pancytopenia, and she died of ARDS, septic shock, and multiorgan failure on her twelfth day of hospitalization. Final histopathological examination of the biopsy specimens showed chronic caseating granulomas with central necrosis (Figure 2), and the tissue culture grew Mycobacterium tuberculosis.

## 3. Discussion

Tuberculous peritonitis is a form of abdominal and/or pelvic TB, which may involve the peritoneum, gastrointestinal tract, lymph nodes, or solid viscera, and constitutes about 1-2% of the total cases. It results either from the spread of adjacent tuberculous disease such as an abdominal or genital focus or hematogenous spread during primary milier pulmonary tuberculosis [9]. TB is a major diagnostic challenge. It has diverse and nonspecific symptoms and laboratory findings and can mimic many other infectious or malignant diseases [10]. Fever, weight loss, and abdominal swelling are the most common symptoms, and abdominal distension, ascites, and abdominal mass are the commonest signs. It has been included in the differential diagnosis of fever of unknown origin, peritoneal carcinomatosis, ovarian cancer, and ascites of portal hypertension or cardiac origin [11].

Pulmonary lesions might not be recognized as TB or the infections might not be evident on chest radiography. Pleural effusion may be the sole radiologic finding in some patients [12]. The most common sonographic and CT findings are ascites, thickening of the viscera (omental, mesenteric, peritoneal, and intestinal), adhesions between viscera, and lymphadenopathy [13]. Laparoscopic findings reported by different authors are typically, exudative, cloudy ascites with multiple whitish nodules or tubercles studding the visceral and parietal peritoneum, filmy extensive adhesions, and omental thickening [14, 15]. Both imaging and operative findings were also evident in our case.

An elevated serum level of Ca-125 is a nonspecific marker of ovarian cancer. It may also be elevated in various gynecological and nongynecological conditions such as tuberculous peritonitis, endometriosis, menstruation, pelvic infection, peritonitis, pancreatitis, hepatitis, nephrotic syndrome, pericarditis, and pneumonia [16]. Several case series of tuberculous peritonitis mimicking ovarian cancer with elevated Ca-125 levels have been reported [17, 18]. However, ovarian cancer is rare before the age of 40, but peritoneal TB most often occurs in patients between the ages of 20 and 40 years [18], as reported in our paper.

There is a controversy regarding the best way to diagnose tuberculous peritonitis. Caseating granulomatous inflammation may be a hallmark of tuberculous peritonitis. For a definite diagnosis, a histological examination is often required. The diagnosis can also be confirmed by the culture and PCR of the infected tissues. However, the yield rate of culturing for Mycobacterium tuberculosis from body fluids is low, and the turnaround time for culturing is about 6 weeks, which may cause a diagnostic and therapeutic delay if the patient actually has ovarian cancer [19]. In a review, 18% of cases with disseminated TB were diagnosed postmortem, compared with 4.8% among those with pulmonary TB [20]. Therefore, preoperative evaluation of body fluids may not provide sufficient evidence before any surgical intervention. Other noninvasive screening tests such as ascitic fluid analysis, adenosine deaminase estimation in ascites and imaging at best can be adjunctive diagnostic tools [13, 21].

Extrapulmonary organ manifestations of TB in particular can be associated with misdiagnosis and fatal outcome [22]. Disseminated TB can cause vital functional disturbances depending on the stage of the disease and the organ manifestation. The mortality from disseminated TB is approximately 20% [23]. ARDS with milier TB has definitely a higher mortality rate. Mortality has been reported to be as high as 100% if there is associated pancytopenia [24]. Other factors associated with mortality include delayed diagnosis and therapy and presence of disseminated intravascular coagulopathy, septic shock, and multiorgan failure. It was estimated that 1-2% of cases with ARDS were associated with disseminated TB [25].

The incidence of TB in pregnancy is not readily available in many countries due to a lot of confounding factors. It is, however, expected that the incidence among pregnant women would be as high as in the general population. The effects of TB on pregnancy may be influenced by many factors, including the severity of the disease, how advanced the pregnancy has gone at the time of diagnosis, the presence of extrapulmonary spread, and HIV coinfection, and the treatment instituted. The worst prognosis is recorded in women in whom a diagnosis of advanced disease is made in the puerperium as well as those with HIV coinfection [5].

In the current report, we presented a rare fatal combination of disseminated tuberculous peritonitis and ARDS following spontaneous abortion. Initial diagnostic workup studies failed to determine the actual disease, and she dramatically died of disease on her twelfth day of hospitalization. This report emphasizes the prognostic significance of pregnancy on TB and the importance of early diagnosis and management. Early administration of antimycobacterial chemotherapy and mechanical ventilation particularly in the presence of ARDS are crucial to optimizing the outcome.
